# The Prognostic Impact of HER2-Low and Menopausal Status in Triple-Negative Breast Cancer

**DOI:** 10.3390/cancers16142566

**Published:** 2024-07-17

**Authors:** Woong Ki Park, Seok Jin Nam, Seok Won Kim, Jeong Eon Lee, Jonghan Yu, Se Kyung Lee, Jai Min Ryu, Byung Joo Chae

**Affiliations:** 1 Division of Breast Surgery, Department of Surgery, Samsung Medical Center, Sungkyunkwan University School of Medicine, Seoul 06351, Republic of Korea; woongki.park@samsung.com (W.K.P.); seokjin.nam@samsung.com (S.J.N.); seokwon1.kim@samsung.com (S.W.K.); jeongeon.lee@samsung.com (J.E.L.); jonghan.yu@samsung.com (J.Y.); sekyung.lee@samsung.com (S.K.L.); jaimin.ryu@samsung.com (J.M.R.); 2 Department of Health Sciences and Technology, Samsung Advanced Institute for Health Science & Technology, Sungkyunkwan University, Seoul 06351, Republic of Korea

**Keywords:** breast cancer, triple negative, HER2-low, oncologic outcomes, menopausal status

## Abstract

**Simple Summary:**

Triple-negative breast cancer (TNBC) is a challenging form of breast cancer known for its aggressive nature and limited treatment options. The DESTINY-Breast 04 trial has demonstrated improved survival outcomes in metastatic human epithelial growth factor receptor 2 (HER2)-low breast cancer patients treated with trastuzumab-deruxtecan (T-Dxd). Since the potential proportion of breast cancer patients that may benefit from these novel agents are reported to be around 39% to 79%, investigating the role of HER2-low has been a topic of interest. By analyzing the outcomes of over 2500 patients, we discovered that those with HER2-low TNBC typically experience better survival rates than HER2-0 TNBC. This difference was especially significant in postmenopausal women. These findings suggest a potential re-evaluation of how TNBC is classified and treated, highlighting the importance of identifying HER2-low expression for improving personalized treatment strategies and patient care.

**Abstract:**

TNBC is noted for its aggressive behavior and poor prognosis. Recently developed HER2 target agents have shown potential benefit even in HER2-low expressing breast cancers. This study retrospectively analyzed 2542 non-metastatic TNBC patients from 2008 to 2020, revealing that 26.0% were HER2-low. Data on demographics, tumor characteristics, pathologic complete response (pCR) rates and disease-free survival (DFS), distant metastasis-free survival (DMFS), overall survival (OS), and breast cancer-specific survival (BCSS) were analyzed. The HER2-low group, compared to the HER2-0 group, showed significantly better DFS, DMFS, OS, BCSS (*p* = 0.0072, *p* = 0.0096, *p* = 0.0180, and *p* = 0.0001, respectively) with older age and higher rates of postmenopausal status (*p* < 0.0001). No significant differences in pCR rates were observed. Multivariate analyses identified HER2 status as a significant prognostic factor for DFS (*p* = 0.048), DMFS (*p* = 0.018), OS (*p* = 0.049), and BCSS (*p* = 0.008). Subgroup analysis revealed that these effects varied with menopausal status, showing more pronounced benefits in postmenopausal women. Our findings suggest that HER2-low TNBC patients exhibit a distinct clinical profile and improved survival compared to HER2-0 TNBC patients, especially in postmenopausal patients. Further research on estrogen and HER2 interaction is needed.

## 1. Introduction

Breast cancer, the most common cancer in women worldwide, is classified into four subtypes with distinct features. TNBC is a subtype with a lack of expression of the estrogen receptor (ER), progesterone receptor (PR), and HER2 [1]. Because of the expression of these markers, ER/PR positive breast cancers and HER2-positive breast cancers can be targeted with endocrine therapy and HER2 target agents [2,3]. On the other hand, TNBC is known to have more aggressive features, and the lack of ER/PR/HER2 expression makes it difficult to target TNBC, therefore presenting a poorer prognosis compared to hormone receptor positive breast cancer [4,5].

On the other hand, the development of HER2 targeting agents have remarkably improved prognosis in HER2 positive breast cancer, and novel agents such as antibody–drug conjugates (ADCs) are showing improved results [3,6,7]. Recent interest has focused on the efficacy of ADCs in treating HER2-low breast cancer, defined as a HER2 immunohistochemistry (IHC) score of 1+ or 2+ with negative in situ hybridization (ISH). Recently, researchers of the DESTINY-Breast 04 trial have reported improved survival outcomes in metastatic HER2-low breast cancer patients treated with T-Dxd [8]. Since the potential proportion of breast cancer patients that may benefit from these novel agents are reported to be around 39% to 79%, investigating the role of HER2-low has been a topic of interest [9,10,11].

Even though many studies have investigated the impact of HER2-low expression on oncologic outcomes, its significance is still unclear. While several studies showed improved survival in patients with HER2-low breast cancer compared to patients with HER2-0 breast cancer [10,12,13,14,15,16], other studies showed no significant difference in survival between the two groups [9,17,18,19].

Previously, we have reported the impact of HER2-low expression in hormone-receptor positive breast cancer. Although HER2 status was a significant prognostic factor, the impact differed among premenopausal and postmenopausal patients [20]. Thus, this study was conducted to verify whether similar results would be obtained in TNBC. The main objective of this study was to compare the oncologic outcomes according to HER2 status and analyze the prognostic impact of HER2-low. Furthermore, we aimed to investigate whether menopausal status affected the prognostic impact of HER2-low. Additionally, we also aimed to investigate whether the response to neoadjuvant chemotherapy (NAC) differed between the two groups.

## 2. Materials and Methods

### 2.1. Study Population

We retrospectively analyzed non-metastatic TNBC patients treated from January 2008 to December 2020 at Samsung Medical Center (SMC). Data were retrieved from our institution’s prospectively collected database. Primary invasive breast cancer cases, negative for ER/PR and HER2, were included. Patients diagnosed with in situ carcinomas, malignancies other than primary invasive breast cancers, stage IV disease at initial diagnosis, and male patients were excluded. Additionally, patients with a follow-up period of less than 12 months after surgery were excluded. However, patients who deceased or experienced recurrence or metastasis within 12 months were included. Overall, a total of 2542 patients were included for analysis.

### 2.2. Data Collection

Data on baseline characteristics, including age at diagnosis, initial menopausal status, BRCA1/2 mutation status, multiplicity of the tumor, tumor size, lymph node metastasis status, nuclear grade (NG), histologic grade (HG), lymphovascular invasion (LVI) status, ER, PR status, HER2 status, and treatment applied such as type of breast/axillary surgery, neoadjuvant and adjuvant chemotherapy, and radiation therapy. Additionally, response to NAC was also recorded.

**Figure 1:**
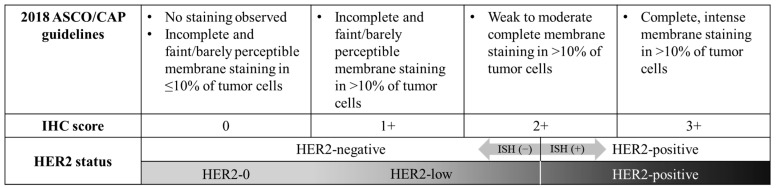
Definition and flowchart of HER2 status. ASCO American Society of Clinical Oncology, CAP College of American Pathologist, IHC immunohistochemistry, ISH in situ hybridization.

To interpret ER, PR, HER2 status, anti-ER, anti-PR, and anti-HER2, monoclonal antibody assays performed on 10% formalin fixed and paraffin-embedded tissue were used. Staining of less than 1% of cells was considered negative for ER and PR [21]. TNBC was immunohistochemically defined as negative for ER/PR and HER2. HER2 status was assessed by the pathologists in the SMC pathology department according to the 2007, 2013, and 2018 American Society of Clinical Oncology (ASCO)/College of American Pathologist (CAP)guidelines. Patients with an IHC score of 0 were defined as HER2-0, and patients with an IHC score of 1+ or 2+ (ISH-negative) were defined as HER2-low [20]. Due to the update in HER2 interpreting guidelines, patients classified as a HER2 IHC score 1+ according to the 2007 ASCO/CAP guidelines would have been classified as HER2 IHC score 0 since the implementation of the 2013 and 2018 ASCO/CAP guidelines. Therefore, to maintain the homogeneity of HER2 classification, these patients were re-classified as HER2-0 in our study. In short, the definition used for interpreting HER2 IHC score 0, 1+, and 2+ was as follows: no staining or incomplete and faint/barely perceptible membrane staining in ≤10% of tumor cells (IHC score 0), incomplete and faint/barely perceptible membrane staining in >10% of tumor cells (IHC score 1+), and weak to moderate complete membrane staining in >10% of tumor cells (IHC score 2+) (Figure 1) [22].

Chemotherapy regimens were administered according to the National Comprehensive Cancer Network (NCCN) guidelines and Korean national health insurance policies [23]. In Korea, ADCs are approved for use in HER2-positive breast cancer patients, and the usage of pembrolizumab in treating TNBC patients was approved in July 2022. Therefore, none of the patients in our study received treatment with ADCs or pembrolizumab. 

The methods and definitions used for cancer staging and menopause were as in the previous study [20]. Menopause was defined as the absence of menstruation for at least 12 consecutive months, and a serum follicle stimulating hormone (FSH) level of 30 mIU/mL or higher. Patient’s recurrent or distant metastasis status and survival data were also collected. All cases were staged according to the 6th or 7th edition of TNM classification by the American Joint Committee on Cancer (AJCC). pCR to NAC was defined as ypTisN0 or ypT0N0. DFS was defined as the interval from date of diagnosis to locoregional recurrence or distant metastasis or censored at the last follow-up date if no events occurred. DMFS was defined as the interval from date of diagnosis to distant metastasis or censored at the last follow-up date if no events occurred. OS was defined as the interval from date of diagnosis to death from any cause or censored at the last follow-up date if no events occurred. BCSS was defined as the interval from date of diagnosis to death caused by breast cancer progression or censored at the last follow-up date if no events occurred.

### 2.3. Statistical Analysis

The Chi-square test or Fisher’s exact test were used for analyzing categorical variables, and we assessed continuous variables using the Mann–Whitney U test. Survival analyses were performed using the Kaplan–Meier method with the *p* values of the log-rank test to assess statistical significance. The Cox regression method was used for univariate and multivariate analyses. Variables showing statistical significance (*p* value < 0.05) in the univariate model were included in the multivariate analysis. All *p* values were two-sided and a *p* value < 0.05 was considered statistically significant. All data analyses were performed with SPSS statistical software program, version 28.0 (SPSS, Chicago, IL, USA) and R statistics, version 4.3.2 (Vienna, Austria; http://www.R-project.org (accessed on 6 July 2024)).

### 2.4. Ethics

This study was approved on 16 January 2024 by the Institutional Review Board (IRB) of our institution (IRB no. SMC 2024-01-057).

## 3. Results

### 3.1. Baseline Characteristics According to HER2 Status

This study evaluated 2542 TNBC patients. A total of 1882 patients (74.0%) were HER2-0, and 660 patients (26.0%) were HER2-low. Table 1 summarized the baseline characteristics of HER2-0 and HER2-low patients. Patients in the HER2-low group were significantly older (median age (interquartile range (IQR)) 48 (41–56) vs. 52 (44–60), *p* < 0.001) and more often postmenopausal (56.4% vs. 46.8%, *p* < 0.001) compared to those in the HER2-0 group. Tumor multiplicity was more common in the HER2-low group, with 19.7% of patients presenting multiple tumors, compared to 15.1% in the HER2-0 group (*p* = 0.005). Significant differences were observed in surgical treatment types; the HER2-low group had higher mastectomy rates (30.8% vs. 22.6%, *p* < 0.001) and lower axillary lymph node dissection (ALND) rates (26.1% vs. 34.4%, *p* < 0.001) compared to the HER2-0 group. The HER2-low group also had a higher rate of patients receiving NAC (39.4% vs. 29.6%, *p* < 0.001) and a lower rate receiving adjuvant chemotherapy (63.2% vs. 73.4%, *p* < 0.001). Radiation therapy was less commonly administered to the HER2-low group (79.8% vs. 86.0%, *p* < 0.001). Pathologically, the HER2-low group exhibited a lower proportion of histologic and nuclear grade III tumors (61.4% vs. 68.8%, *p* < 0.001 and 69.5% vs. 75.2%, *p* = 0.001, respectively), as well as a lower incidence of LVI (18.9% vs. 24.3%, *p* < 0.001).

### 3.2. Oncologic Outcomes According to HER2 Status

**Figure 2:**
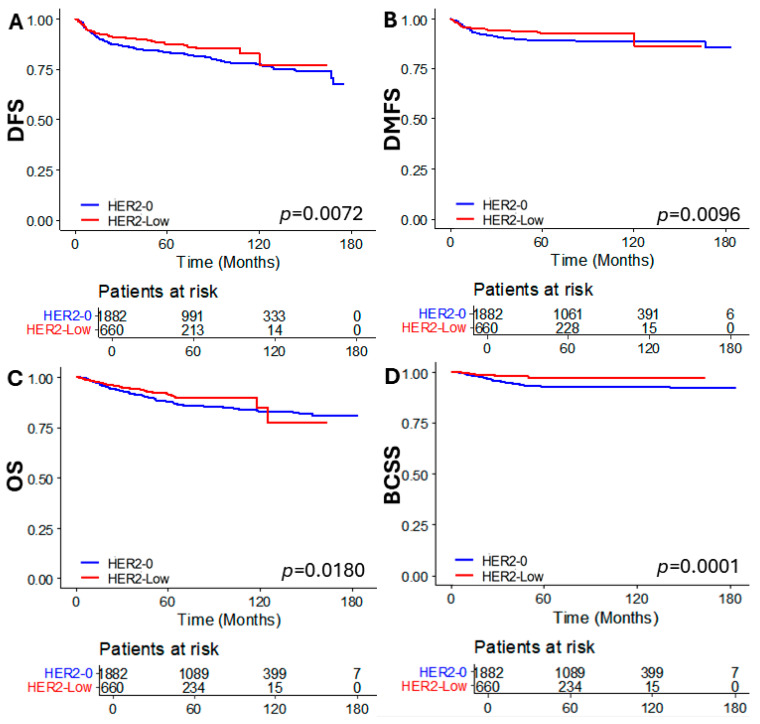
Kaplan–Meier curves of (**A**) DFS, (**B**) DMFS, (**C**) OS, and (**D**) BCSS according to HER2 status. DFS, disease free survival; DMFS, distant metastasis free survival; OS, overall survival; and BCSS, breast cancer specific survival.

The median follow-up time of the total study population was 62 months (IQR: 42–105), 70 months (IQR 45–113) for the HER2-0 group, and 52 months (IQR 37–71) for the HER2-low group. There were significant differences in DFS, DMFS, OS, and BCSS between the HER2-low and HER2-0 group, with better survival outcomes observed in the HER2-low group (*p* = 0.0072, *p* = 0.0096, *p* = 0.0180, and *p* = 0.0001, respectively) (Figure 2).

**Figure 3:**
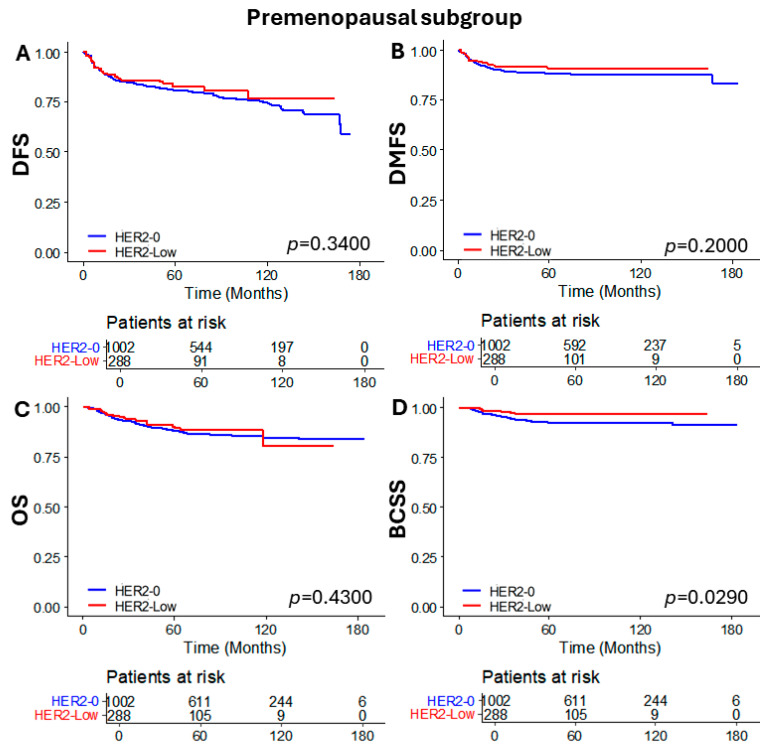
Kaplan–Meier curves of (**A**) DFS, (**B**) DMFS, (**C**) OS, and (**D**) BCSS according to HER2 status in the premenopausal subgroup. DFS, disease free survival; DMFS, distant metastasis free survival; OS, overall survival; and BCSS, breast cancer specific survival.

**Figure 4:**
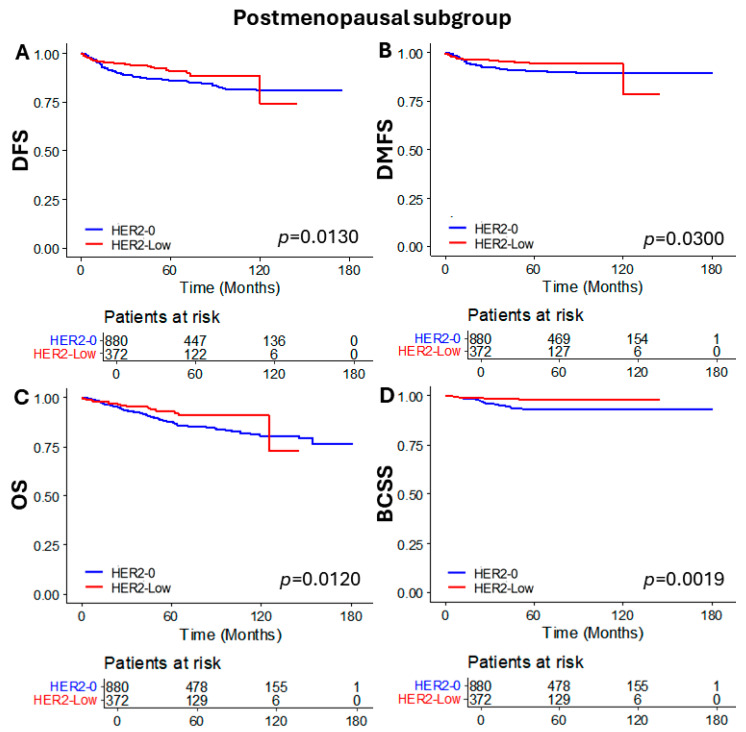
Kaplan–Meier curves of (**A**) DFS, (**B**) DMFS, (**C**) OS, and (**D**) BCSS according to HER2 status in the postmenopausal subgroup. DFS, disease free survival; DMFS, distant metastasis free survival; OS, overall survival; and BCSS, breast cancer specific survival.

To evaluate whether these differences were consistent in both premenopausal and postmenopausal patient groups, we analyzed the oncologic outcomes based on HER2 status in each menopausal subgroup. In the premenopausal subgroup, DFS, DMFS, and OS were not significantly different according to HER2 status. However, significantly better BCSS was observed in the HER2-low group compared to the HER2-0 group (*p* = 0.0290) (Figure 3). On the other hand, significant differences were observed in the postmenopausal subgroup. The HER2-low group demonstrated superior outcomes for DFS (*p* = 0.0130), DMFS (*p* = 0.0300), OS (*p* = 0.0120), and particularly for BCSS, which showed a markedly significant difference (*p* = 0.0019) (Figure 4).

### 3.3. Factors Associated with Oncologic Outcomes

Univariate and multivariate analysis was performed to find out the significant factors associated with DFS, DMFS, OS, and BCSS (Table 2 and Table 3). The number of patients that experienced recurrence, distant metastasis, and death are described in the Supplementary Table S1. In summary, a total of 436 recurrences (190 locoregional recurrences, 172 distant metastases, and 74 both locoregional and distant metastases), and 308 mortalities (139 breast cancer related mortalities and 169 mortalities of other causes) were observed. 

The analysis of DFS revealed several factors significantly associated with outcomes both in univariate and multivariate models. Factors such as HER2 status, age, breast surgery type, axilla surgery type, NAC, tumor size and LVI showed significant associations. HER2-low (HR = 0.761, *p* = 0.048) and age (HR = 0.980, *p* = 0.009) were protective factors in the multivariate model. A mastectomy, ALND, and NAC were associated with poorer DFS (HR = 1.573, *p* < 0.001, HR = 2.099, *p* < 0.001, and HR = 1.872, *p* < 0.001, respectively), likely indicating it was performed on more advanced cases. LVI and larger tumor size (>2 cm) also negatively impacted DFS (HR = 2.244, *p* < 0.001 and HR = 1.393, *p* = 0.029, respectively).

Similar results were notable for DMFS. As in DFS, HER2-low (HR = 0.647, *p* = 0.018) was a protective factor, and mastectomy (HR = 2.149, *p* < 0.001), ALND (HR = 2.904, *p* < 0.001), and NAC (HR = 2.030, *p* < 0.001) were significantly associated with worse DMFS. Additionally, larger tumor size (HR = 2.019, *p* = 0.004) and LVI (HR = 1.925, *p* < 0.001) had a negative impact on DMFS. Compared to the DFS model, age was not a significant factor associated with DMFS.

OS was significantly influenced by HER2 status, the type of breast and axillary surgeries performed, tumor size, LVI, and adjuvant chemotherapy. HER2-low status and receiving adjuvant chemotherapy improved survival outcomes in the multivariate model (HR = 0.725, *p* = 0.049 and HR = 0.566, *p* = 0.002). Mastectomy, ALND, larger tumor size, and LVI were linked to worse OS.

BCSS analyses revealed strong associations with surgical treatment type, LVI, and tumor size. HER2-low status again showed a significant protective effect (HR = 0.476, *p* = 0.008). Extensive surgeries like mastectomy and especially ALND correlated with poorer outcomes (HR = 1.494, *p* = 0.050 and HR = 3.437, *p* < 0.001). The presence of LVI and larger tumor sizes also worsened BCSS.

Overall, the presence of LVI, larger tumor size, and receiving mastectomy and ALND were factors of poorer survival along all survival analyses. On the other hand, HER2-low status consistently emerged as a significant protective factor in all survival outcomes. 

### 3.4. Subgroup Analysis of Patients Who Received NAC

**Figure 5:**
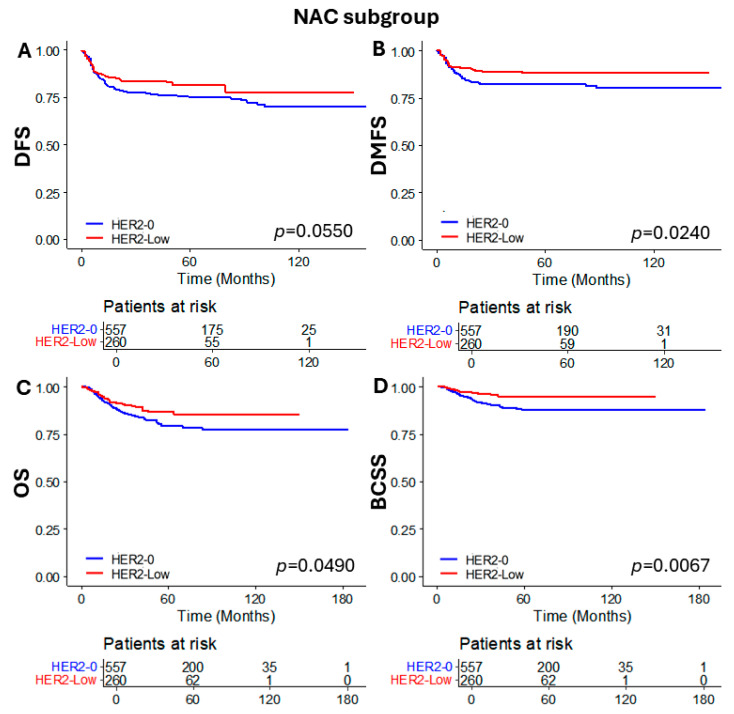
Kaplan–Meier curves of (**A**) DFS, (**B**) DMFS, (**C**) OS, and (**D**) BCSS according to HER2 status in the NAC subgroup. DFS, disease free survival; DMFS, distant metastasis free survival; OS, overall survival; BCSS, breast cancer specific survival; and NAC, neoadjuvant chemotherapy.

To compare oncologic outcomes and response to NAC, we performed a subgroup analysis for patients who received NAC according to HER2 status. The chemotherapy regimens were administered according to the NCCN guidelines and Korean national health insurance policies (for example, dose-dense AC (doxorubicin/cyclophosphamide) preceded by weekly paclitaxel, AC followed by docetaxel, TC (docetaxel and cyclophosphamide), TAC (docetaxel/doxorubicin/cyclophosphamide), paclitaxel + carboplatin, or docetaxel + carboplatin) [23]. Additionally, none of the patients in this study received pembrolizumab because its use in TNBC was approved in 2022. When comparing the baseline characteristics, only age and incidence of LVI were different between the two groups (Table 4). Other than these factors, the two groups showed similar characteristics. Additionally, no significant difference in pCR rate was observed (26.9% vs. 29.1%, *p* = 0.523). However, when comparing the oncologic outcomes, DMFS, OS, and BCSS were significantly better in the HER2-low group (*p* = 0.0240, *p* = 0.0490, and *p* = 0.0067, respectively). Difference in DFS was not statistically significant (*p* = 0.0550) (Figure 5). 

## 4. Discussion

In our previous study of hormone-receptor-positive breast cancer patients, we reported significantly improved oncologic outcomes in the HER2-low group compared to the HER2-0 group (median follow-up period of 64 months). However, these findings differed according to menopausal status. Also, HER2 status was an independent predictor of DFS, OS, and BCSS [20]. With a median follow-up period of 62 months, this study demonstrated that similar results were consistent in the TNBC patients. Improved oncologic outcomes were observed in the HER2-low group, and HER2 status was an independent predictor of DFS, DMFS, OS, and BCSS. The impact of HER2-low differed according to menopausal status. Although significant differences in oncologic outcomes according to HER2 status were consistent in the NAC subgroup, there were no differences in pCR rates based on HER2 status.

Previous studies have reported varying proportions of HER2-low in TNBC, ranging from 17.6% to 66.3% [9,10,12,24,25]. In our study, HER2-low proportion was 26.0%. 

Some notable differences were noted in baseline characteristics between the HER2-low group and HER2-0 group. The HER2-low group had a higher proportion of postmenopausal patients and older age compared to the HER2-0 group. In addition, patients presenting with multiple tumors were more frequent in the HER2-low group. This may have been one of the factors that affected the higher mastectomy rate in the HER2-low group. Furthermore, due to the higher rate of mastectomy and SLNB, fewer patients received adjuvant radiation therapy in the HER2-low group. While there were no differences in tumor size and lymph node metastasis, the HER2-low group had fewer tumors with high grade and LVI. These characteristics were comparable to other previous studies that investigated HER2-low TNBC patients. In a retrospective study of 296 non-metastatic TNBC patients, HER2-low patients were older, had a lower HG, and had an apocrine-like molecular feature compared to the HER2-0 group [26]. Within the Korean nationwide study, HER2-low TNBC patients were older and had tumors with a lower Ki-67 index [10]. Similarly, older age and smaller proportion of T3 tumors were observed in a retrospective study that included 466 TNBC patients [9].

The impact of HER2-low on oncologic outcomes was significant in our study, with favorable results in the HER2-low group. Several past studies, including a retrospective study of 4918 breast cancer patients, described that HER2-low status had no prognostic impact on oncologic outcomes [9,17,26,27,28]. On the contrary, other studies showed that HER2-low was a significant prognostic factor [10,11,12,13,14,15,16,19]. Furthermore, discordance in the expression of HER2 status were observed between the primary tumor and recurred or metastatic tissue [29]. Considering these findings, whether HER2-low status truly has a prognostic value seems unclear. Nevertheless, it seems important to reassess the tumor subtype including HER2 status in the recurrent/metastatic situation because the HER2 status may have dynamically changed, and the patient could benefit from targetable agent such as T-Dxd. 

Currently, there are no concrete explanations on why HER2-low breast cancer has better outcomes compared to HER2-0 breast cancer. Using single-cell RNA sequencing, Hu et al. revealed that HER2-0 TNBC had a more active immune microenvironment and higher levels of immunotherapeutic biomarkers compared to HER2-low TNBC [25]. Tan et al. reported that ERBB2 copy number variant (CNV) score was an independent prognostic factor for recurrence free survival [12]. Also, Agostinetto et al. demonstrated that ERBB2 expression correlated with HER2 IHC scores [17]. An international multicenter cohort study of 28,280 non-metastatic breast cancer patients described that better recurrence free survival and OS of HER2-low did not correlate with HER2 IHC score and mainly resulted from the HER2 IHC1+ subgroup [12]. A study that re-evaluated HER2 staining status in 281 breast cancer cases identified inter-observer variations in evaluating HER2 IHC staining, specifically for scores IHC 0 and IHC 1+ [24]. This poor reproducibility of HER2 IHC evaluation is a big hurdle in clearly defining HER2-low status, with inter-observer variations up to 75% reported when reassessed [30]. Therefore, setting the boundary (especially the lower limit of HER2-low) for selecting potential patients that may benefit from treatment with T-Dxd is challenging. Further dividing HER2-0 into HER2-null and IHC > 0 to <1+ has been suggested, and clinical trials are ongoing to see the efficacy of T-Dxd on this population [31,32,33]. Future studies on researching the tumor microenvironment and biomarkers of HER2-low breast cancer are necessary. In summary, new assays for a more precise evaluation of HER2 status seems necessary. Furthermore, as we await the results of ongoing clinical trials, re-defining the lower-limit of HER2-low may be needed in the future.

In addition to the improvement and development of tailored systemic treatment, there had been efforts to investigate the feasibility of tailored surgical treatment according to different subtypes in recurrent cases. Gentile et al. revealed that a second breast conserving surgery may be feasible in recurrent small-sized HER2-positive cases or TNBCs [34]. When combined with the novel target agents, this may improve both survival and the quality of life of selected patients.

Achieving pCR after NAC is an important prognostic factor in breast cancer treatment [35]. Therefore, pCR rate is measured as one of the primary outcomes in many studies including those regarding the HER2-low group [11,18,36,37,38,39,40]. The pCR rate in the HER2-low group ranged from 11.86% to 51%, while the HER2-0 group ranged from 15.2% to 52% [11,36,38,39,40]. In a retrospective nationwide study including 20,029 TNBC patients, Li, H. et al. reported significantly lower pCR rates in the HER2-low group compared to the HER2-0 group [11]. On the contrary, Denkert et al., conducted a pooled analysis of four prospective neoadjuvant clinical trials and found no difference in the pCR rates according to HER2 status in TNBC patients [15]. Interestingly, better oncologic outcomes were observed in the HER2-low TNBC group in both studies. In our study, the pCR rate did not differ between HER2-low and HER2-0 group (26.9% vs. 29.1%, respectively). Jacot et al. reported that HER2-low TNBC expressed more apocrine-like molecular features with lower HG compared to HER2-0 TNBC [26]. These relatively less-aggressive tumor characteristics of HER2-low breast cancer may be the reason why HER2-low breast cancer has favorable prognosis even though the response to systemic treatment is not different compared to HER2-0 breast cancer.

Our study has limitations. Due to the retrospective nature of this study, incomplete patient data were included. Therefore, the possibility of other confounding variables of measured or unmeasured factors exists. Furthermore, since it was a single center study, we only included Korean TNBC patients. Therefore, further multi-national study results are necessary to generalize our results on a global basis. We included patients from 2008 to 2020. Between this time interval, guidelines on treatment and management have changed, and thus may have affected the oncologic outcomes. 

To our knowledge, this is the largest single center study examining the impact of HER2-low status on oncologic outcomes in TNBC patients. Moreover, data were extracted from our prospectively collected database, ensuring the homogeneity of the treatment applied. Another strength of our study is the reclassification of patients from HER2 IHC score 1+ to HER2-0 according to the updated ASCO/CAP guidelines, ensuring homogeneity in HER2 status classification. Compared to the other studies, our previous study and current study revealed that the impact of HER2-low on oncologic outcomes differed according to menopausal status [20]. In a study that analyzed the effect of menopausal status in different breast cancer subtypes, the authors reported a higher proportion of postmenopausal patients in the TNBC subtype and a tendency to have favorable outcomes compared to premenopausal patients [41]. Our study demonstrated a higher proportion of postmenopausal patients in the HER2-low TNBC group. Also, the expression of HER2-low seemed to have a positive association with ER expression [42]. Considering the lower estrogen level and favorable prognosis of postmenopausal patients compared to premenopausal women, future studies are needed to investigate the possible crosstalk between estrogen and HER2 expression.

## 5. Conclusions

Despite similar rates of pCR, our study revealed that HER2-low status was an independent predictor of improved DFS, DMFS, OS, and BCSS compared to HER2-0 in TNBC. However, the prognostic impact differed between menopausal status, especially benefitting postmenopausal patients. Therefore, assessing HER2 status along with menopausal status may help predict prognosis in TNBC patients. Updates in interpreting HER2 expression seem necessary to precisely define the boundaries of HER2-low expression. Future studies to discover the crosstalk between hormone levels and HER2 expression are required.

## Figures and Tables

**Table 1:** Baseline characteristics according to HER2 expression.

	HER2-0	HER2-Low	*p*-Value
**Clinical variables**			
Total no. (%)	1882 (74.0)	660 (26.0)	
Age			
Median (IQR)	48 (41–56)	52 (44–60)	<0.001
Menopausal status, no. (%)			<0.001
Pre	1002 (53.2)	288 (43.6)	
Post	880 (46.8)	372 (56.4)	
*BRCA 1/2* mutation, no. (%)			0.510
Not detected	1736 (92.2)	614 (93.0)	
Detected	146 (7.8)	46 (7.0)	
Laterality			0.888
Unilateral cancer	1866 (99.1)	654 (99.1)	
Bilateral cancer	16 (0.9)	6 (0.9)	
Multiplicity			0.005
No	1567 (83.3)	514 (77.9)	
Yes	285 (15.1)	130 (19.7)	
Unknown	30 (1.6)	16 (2.4)	
Breast surgery, no. (%)			<0.001
BCS	1457 (77.4)	457 (69.2)	
Mastectomy	425 (22.6)	203 (30.8)	
Axilla surgery, no. (%)			<0.001
SLNB	1235 (65.6)	488 (73.9)	
ALND	647 (34.4)	172 (26.1)	
Neoadjuvant chemotherapy, no. (%)			<0.001
No	1325 (70.4)	400 (60.6)	
Yes	557 (29.6)	260 (39.4)	
Adjuvant chemotherapy, no. (%)			<0.001
No	501 (26.6)	243 (36.8)	
Yes	1381 (73.4)	417 (63.2)	
Radiation therapy, no. (%)			<0.001
No	264 (14.0)	133 (20.2)	
Yes	1618 (86.0)	527 (79.8)	
**Pathologic variables**			
Tumor size			0.109
≤2 cm	712 (37.8)	273 (41.4)	
>2 cm	1170 (62.2)	387 (58.6)	
Lymph node metastasis			0.504
Negative	1069 (56.8)	365 (55.3)	
Positive	813 (43.2)	295 (44.7)	
Histologic grade			<0.001
≤II	420 (22.3)	190 (28.8)	
>II	1295 (68.8)	405 (61.4)	
Unknown	167 (8.9)	65 (9.8)	
Nuclear grade			0.001
≤II	313 (16.6)	147 (22.3)	
>II	1416 (75.2)	459 (69.5)	
Unknown	153 (8.2)	54 (8.2)	
Lymphovascular invasion			<0.001
No	881 (46.8)	444 (67.3)	
Yes	457 (24.3)	125 (18.9)	
Unknown	544 (28.9)	91 (13.8)	

IQR, interquartile range; BCS, breast conserving surgery; SLNB, sentinel lymph node biopsy; ALND, axillary lymph node dissection.

**Table 2:** Univariate and multivariate Cox models regarding DFS and DMFS.

	Univariate Model	*p*-Value	Multivariate Model	*p*-Value
	HR (95% CI)		HR (95% CI)	
**Disease Free Survival**				
HER-2 status (0 vs. low)	0.715 (0.559–0.915)	0.008	0.761 (0.581–0.997)	0.048
Age	0.977 (0.968–0.985)	<0.001	0.980 (0.965–0.995)	0.009
Menopausal status (pre- vs. post-)	0.628 (0.518–0.763)	<0.001	0.909 (0.649–1.272)	0.577
*BRCA1/2* mutation status (No vs. Yes)	1.302 (0.950–1.784)	0.100		
Laterality (uni- vs. bilateral)	1.373 (0.568–3.316)	0.481		
Multiplicity (single vs. multiple)	1.290 (1.017–1.636)	0.036	0.830 (0.629–1.094)	0.186
Breast Surgery (BCS vs. Mastectomy)	2.040 (1.677–2.481)	<0.001	1.573 (1.236–2.002)	<0.001
Axilla Surgery (SLNB vs. ALND)	2.794 (2.311–3.377)	<0.001	2.099 (1.553–2.836)	<0.001
NAC (No vs. Yes)	2.070 (1.705–2.513)	<0.001	1.872 (1.327–2.640)	<0.001
Adjuvant CTx (No vs. Yes)	0.602 (0.494–0.734)	<0.001	0.778 (0.570–1.060)	0.112
Adjuvant RT (No vs. Yes)	0.861 (0.670–1.108)	0.245		
Tumor size (≤2 cm vs. >2 cm)	2.017 (1.673–2.573)	<0.001	1.393 (1.035–1.875)	0.029
LN metastasis (No vs. Yes)	2.115 (1.747–2.559)	<0.001	0.777 (0.566–1.066)	0.118
HG (≤GrII vs. >GrII)	1.073 (0.860–1.339)	0.532		
NG (≤GrII vs. >GrII)	1.128 (0.879–1.448)	0.345		
LVI (No vs. Yes)	3.141 (2.529–3.901)	<0.001	2.244 (1.746–2.884)	<0.001
**Distant Metastasis Free Survival**				
HER-2 status (0 vs. low)	0.652 (0.470–0.903)	0.010	0.647 (0.451–0.929)	0.018
Age	0.984 (0.972–0.995)	0.005	0.989 (0.970–1.009)	0.281
Menopausal status (pre- vs. post-)	0.721 (0.559–0.930)	0.012	0.861 (0.559–1.326)	0.497
*BRCA1/2* mutation status (No vs. Yes)	0.775 (0.460–1.306)	0.339		
Laterality (uni- vs. bilateral)	1.475 (0.472–4.608)	0.503		
Multiplicity (single vs. multiple)	1.069 (0.766–1.491)	0.696		
Breast Surgery (BCS vs. Mastectomy)	3.258 (2.536–4.185)	<0.001	2.149 (1.586–2.913)	<0.001
Axilla Surgery (SLNB vs. ALND)	5.113 (3.901–6.702)	<0.001	2.904 (1.912–4.410)	<0.001
NAC (No vs. Yes)	2.779 (2.159–3.575)	<0.001	2.030 (1.334–3.088)	<0.001
Adjuvant CTx (No vs. Yes)	0.515 (0.399–0.664)	<0.001	0.942 (0.647–1.372)	0.757
Adjuvant RT (No vs. Yes)	0.849 (0.611–1.180)	0.331		
Tumor size (≤2 cm vs. >2 cm)	3.569 (2.544–5.009)	<0.001	2.019 (1.259–3.238)	0.004
LN metastasis (No vs. Yes)	3.623 (2.745–4.781)	<0.001	0.929 (0.595–1.452)	0.748
HG (≤GrII vs. >GrII)	0.968 (0.725–1.293)	0.826		
NG (≤GrII vs. >GrII)	0.913 (0.668–1.248)	0.568		
LVI (No vs. Yes)	3.855 (2.891–5.142)	<0.001	1.925 (1.382–2.681)	<0.001

DFS, disease free survival; DMFS, distant metastasis free survival; BCS, breast conserving surgery; SLNB, sentinel lymph node biopsy; ALND, axillary lymph node dissection; NAC, neoadjuvant chemotherapy; CTx, chemotherapy; RT, radiation therapy; LN, lymph node; HG, histologic grade; NG, nuclear grade; LVI, lymphovascular invasion.

**Table 3:** Univariate and multivariate Cox models regarding OS and BCSS.

	Univariate Model	*p*-Value	Multivariate Model	*p*-Value
	HR (95% CI)		HR (95% CI)	
**Overall Survival**				
HER-2 status (0 vs. low)	0.698 (0.517–0.943)	0.019	0.725 (0.526–0.999)	0.049
Age	1.006 (0.996–1.016)	0.271		
Menopausal status (pre- vs. post-)	1.052 (0.841–1.316)	0.656		
*BRCA1/2* mutation status (No vs. Yes)	0.805 (0.512–1.267)	0.349		
Laterality (uni- vs. bilateral)	1.953 (0.807–4.728)	0.138		
Multiplicity (single vs. multiple)	1.116 (0.834–1.494)	0.461		
Breast Surgery (BCS vs. Mastectomy)	2.706 (2.158–3.392)	<0.001	1.458 (1.074–1.978)	0.016
Axilla Surgery (SLNB vs. ALND)	4.304 (3.391–5.462)	<0.001	2.697 (1.858–3.914)	<0.001
NAC (No vs. Yes)	2.262 (1.799–2.843)	<0.001	1.440 (0.953–2.174)	0.083
Adjuvant CTx (No vs. Yes)	0.468 (0.372–0.587)	<0.001	0.566 (0.395–0.812)	0.002
Adjuvant RT (No vs. Yes)	0.706 (0.535–0.933)	0.014	0.726 (0.501–1.054)	0.092
Tumor size (≤2 cm vs. >2 cm)	3.075 (2.311–4.093)	<0.001	2.070 (1.407–3.045)	<0.001
LN metastasis (No vs. Yes)	2.942 (2.322–3.729)	<0.001	0.880 (0.598–1.294)	0.516
HG (≤GrII vs. >GrII)	1.052 (0.962–1.152)	0.266		
NG (≤GrII vs. >GrII)	1.077 (0.971–1.193)	0.161		
LVI (No vs. Yes)	3.575 (2.768–4.618)	<0.001	2.229 (1.657–2.997)	<0.001
**Breast cancer Specific Survival**				
HER-2 status (0 vs. low)	0.368 (0.215–0.630)	<0.001	0.476 (0.274–0.827)	0.008
Age	0.982 (0.967–0.997)	0.021	0.983 (0.966–1.001)	0.065
Menopausal status (pre- vs. post-)	0.757 (0.540–1.060)	0.105		
*BRCA1/2* mutation status (No vs. Yes)	0.626 (0.293–1.339)	0.227		
Laterality (uni- vs. bilateral)	1.710 (0.423–6.907)	0.451		
Multiplicity (single vs. multiple)	1.117 (0.724–1.722)	0.617		
Breast Surgery (BCS vs. Mastectomy)	2.640 (1.887–3.694)	<0.001	1.494 (0.999–2.234)	0.05
Axilla Surgery (SLNB vs. ALND)	5.907 (4.067–8.578)	<0.001	3.437 (1.911–6.179)	<0.001
NAC (No vs. Yes)	2.448 (1.752–3.419)	<0.001	1.551 (0.861–2.795)	0.144
Adjuvant CTx (No vs. Yes)	0.492 (0.351–0.688)	<0.001	0.652 (0.378–1.122)	0.123
Adjuvant RT (No vs. Yes)	1.009 (0.634–1.605)	0.971		
Tumor size (≤2 cm vs. >2 cm)	4.981 (2.999–8.274)	<0.001	3.107 (1.549–6.230)	0.001
LN metastasis (No vs. Yes)	3.299 (2.296–4.739)	<0.001	0.797 (0.441–1.440)	0.452
HG (≤GrII vs. >GrII)	1.117 (0.972–1.283)	0.118		
NG (≤GrII vs. >GrII)	1.109 (0.950–1.295)	0.191		
LVI (No vs. Yes)	4.948 (3.320–7.375)	<0.001	2.527 (1.609–3.970)	<0.001

OS, overall survival; BCSS, breast cancer specific survival; BCS, breast conserving surgery; SLNB, sentinel lymph node biopsy; ALND, axillary lymph node dissection; NAC, neoadjuvant chemotherapy; CTx, chemotherapy; RT, radiation therapy; LN, lymph node; HG, histologic grade; NG, nuclear grade; LVI, lymphovascular invasion.

**Table 4:** Baseline characteristics according to HER2 status in the NAC subgroup.

	HER2-0	HER2-Low	*p*-Value
**Clinical variables**			
Total no.	557 (68.2)	260 (31.8)	
Age			0.014
Median (IQR)	46.00 (38–53)	48.00 (40–55.75)	
Menopausal status, no. (%)			0.066
Pre menopause	342 (61.4)	142 (54.6)	
Post menopause	215 (38.6)	118 (45.4)	
*BRCA 1/2* mutation, no. (%)			0.906
Not detected	507 (91.0)	236 (90.8)	
Detected	50 (9.0)	24 (9.2)	
Laterality			1.000
Unilateral cancer	554 (99.5)	259 (99.6)	
Bilateral cancer	3 (0.5)	1 (0.4)	
Multiplicity			0.490
No	442 (83.7)	209 (85.7)	
Yes	86 (16.3)	35 (14.3)	
Unknown			
Breast surgery, no. (%)			0.647
BCS	368 (66.1)	176 (67.7)	
Mastectomy	189 (33.9)	84 (32.3)	
Axilla surgery, no. (%)			0.245
SLNB	295 (53.0)	149 (57.3)	
ALND	262 (47.0)	111 (42.7)	
Adjuvant chemotherapy, no. (%)			0.339
No	385 (69.1)	171 (65.8)	
Yes	172 (30.9)	89 (34.2)	
Radiation therapy, no. (%)			0.395
No	53 (9.5)	20 (7.7)	
Yes	504 (90.5)	240 (92.3)	
**Pathologic variables**			
Clinical T stage			0.071
1	39 (7.0)	20 (7.7)	
2	377 (67.7)	190 (73.1)	
3	102 (18.3)	43 916.5)	
4	39 (7.0)	7 (2.7)	
Clinical N stage			0.537
0	81 (14.5)	43 (16.5)	
1	212 (38.1)	108 (41.5)	
2	162 (29.1)	67 (25.8)	
3	102 (18.3)	42 (16.2)	
Histologic grade			0.760
≤II	126 (31.0)	65 (32.2)	
>II	281 (69.0)	137 (67.8)	
Unknown			
Nuclear grade			0.738
≤II	69 (16.5)	32 (15.5)	
>II	349 (83.5)	175 (84.5(	
Unknown			
Lymphovascular invasion			0.007
No	236 (42.4)	144 (55.4)	
Yes	132 (23.7)	47 (18.1)	
Unknown	189 (33.9)	69 (26.5)	
Pathologic response			0.523
pCR	162 (29.1)	70 (26.9)	
non-pCR	395 (70.9)	190 (73.1)	

IQR, interquartile range; NAC, neoadjuvant chemotherapy; BCS, breast conserving surgery; SLNB, sentinel lymph node biopsy; ALND, axillary lymph node dissection; pCR, pathologic complete response.

## Data Availability

The datasets used and/or analyzed during the current study are available from the corresponding author on reasonable request.

## Supplementary Materials

The following supporting information can be downloaded at: https://www.mdpi.com/article/10.3390/cancers16142566/s1. Table S1. Number of recurrences and deaths occurred according to HER2 status.

## Abbreviations

ADCs, antibody–drug conjugates; AJCC, American Joint Committee on Cancer; ALND, axillary lymph node dissection; ASCO, American Society of Clinical Oncology; BCSS, Breast cancer specific survival; CAP, College of American Pathologist; CNV, copy number variant; DFS, disease free survival; DMFS, distant metastasis free survival; ER, estrogen receptor; FSH, follicle-stimulating hormone; HER2, human epithelial growth factor receptor 2; HG, histologic grade; IHC, immunohistochemistry; IRB, Institutional Review Board; ISH, in situ hybridization; LVI, lymphovascular invasion; NAC, neoadjuvant chemotherapy; NCCN, National Comprehensive Cancer Network; NG, nuclear grade; OS, overall survival; pCR, pathologic complete response; PR, progesterone receptor; SMC, Samsung Medical Center; T-Dxd, trastuzumab-deruxtecan; TNBC, triple-negative breast cancer.
