# Supplementary material for: The Prognostic Impact of HER2-Low and Menopausal Status in Triple-Negative Breast Cancer
Source: Cancers (Basel). 2024 Jul 17;16(14):2566. doi: 10.3390/cancers16142566 (PMC11274797; doi:10.3390/cancers16142566)
Supplement: Supplementary file 1 [file cancers-16-02566-s001.zip › cancers-3068218-supplementary.pdf]

Supplemental Table S1. Number of recurrences and deaths occurred according to HER2 status.

	HER2-0, no. (%)	HER2-low, no. (%)
**Recurrence**		
Locoregional recurrence	156 (43.6)	34 (43.6)
Distant metastasis	136 (38.0)	36 (46.2)
Locoregional recurrence & Distant metastasis	66 (18.4)	8 (10.2)
**Death**		
Breast cancer related death	124 (48.4)	15 (28.8)
Others	132 (51.6)	37 (71.2)
